# Conjugated Electron Donor–Acceptor Hybrid Polymeric Carbon Nitride as a Photocatalyst for CO_2_ Reduction

**DOI:** 10.3390/molecules24091779

**Published:** 2019-05-08

**Authors:** Asif Hayat, Mati Ur Rahman, Iltaf Khan, Javid Khan, Muhammad Sohail, Humaira Yasmeen, Shu-yuan Liu, Kezhen Qi, Wenxiu Lv

**Affiliations:** 1 Institute of Catalysis for Energy and Environment, College of Chemistry and Chemical Engineering, Shenyang Normal University, Shenyang 110034, China; 15164052089@163.com; 2 College of Chemistry, Fuzhou University, Fuzhou 350002, China; asifncp11@yahoo.com (A.H.); matiurrahman617@yahoo.com (M.U.R.); 3 Key Laboratory of Functional Inorganic Material Chemistry, School of Chemistry and Materials Science, Heilongjiang University, Harbin 158308, China; iltafkhanpakistan@gmail.com; 4 MOE Key Laboratory of Bioinorganic and Synthetic Chemistry, Key Laboratory of Environment and Energy Chemistry of Guangdong Higher Education Institutes, School of Chemistry, Sun Yat-Sen University, Guangzhou 510275, China; javidchemist@yahoo.com; 5 Institute for Advanced Study, Shenzhen University, Shenzhen 518060, China; sohailncp@gmail.com; 6 Key Laboratory of Bio-Based Material Science and Technology, Ministry of Education, Northeast Forestry University, Harbin 150040, China; humairanefu@yahoo.com; 7 Department of pharmacology, Shenyang Medical College, Shenyang 110034, China; 8 Key Laboratory for Photonic and Electronic Bandgap Materials, Ministry of Education, School of Physics and Electronic Engineering, Harbin Normal University, Harbin 150025, China

**Keywords:** polymeric carbon nitride, donor-acceptor, photocatalysis, CO_2_ reduction, DFT calculation

## Abstract

This work incorporates a variety of conjugated donor-acceptor (DA) co-monomers such as 2,6-diaminopurine (DP) into the structure of a polymeric carbon nitride (PCN) backbone using a unique nanostructure co-polymerization strategy and examines its photocatalytic activity performance in the field of photocatalytic CO_2_ reduction to CO and H_2_ under visible light irradiation. The as-synthesized samples were successfully analyzed using different characterization methods to explain their electronic and optical properties, crystal phase, microstructure, and their morphology that influenced the performance due to the interactions between the PCN and the DPco-monomer. Based on the density functional theory (DFT) calculation result, pure PCN and CNU-DP_15.0_ trimers (interpreted as incorporation of the co-monomer at two different positions) were extensively evaluated and exhibited remarkable structural optimization without the inclusion of any symmetry constraints (the non-modified sample derived from urea, named as CNU), and their optical and electronic properties were also manipulated to control occupation of their respective highest occupied molecular orbital (HOMO) and lowest unoccupied molecular orbital (LUMO). Also, co-polymerization of the donor–acceptor 2,6-diamino-purine co-monomer with PCN influenced the chemical affinities, polarities, and acid–base functions of the PCN, remarkably enhancing the photocatalytic activity for the production of CO and H_2_ from CO_2_ by 15.02-fold compared than that of the parental CNU, while also improving the selectivity.

## 1. Introduction

The excessive use of fossil fuels and the increasing concentrations of the resulting pollutants exert a negative impact on human life, the environment and energy resources in the form of global warming. Intensive endeavors are ongoing to meet the challenges of increasing future demand for fuels and the problems caused by their utilization and need for conservation due to excess pressure on the world energy infrastructure [1,2,3]. Enormous efforts have been fueled by scientists to provide sustainable energy sources, such as solar cells, fuel cells, and biofuels, that are can meet the future demand for competitive energy. Importantly, the energy supplied by these energy sources is often unpredictable, and solar cells have been demonstrated to be the most reliable after numerous endeavors [4,5]. The solar energy coming from the sun has enormous potential and is considered a possibly optimal solution to the problem of fossil fuel depletion, but solar energy cannot be used directly, and it must be combined with a carbon-neutral energy source which is a challenge as the system will be solar energy driven, which is an intermittent process. One technology for solving this global issue is the conversion of solar energy into chemical energy that can be stored for later use.

In this regard, the use of conjugated polymer semiconductors is of great current interest as it can economically convert sunlight into chemical energy using different compounds [6,7], contrary to other, more common semiconductors [8]. The dominant factor that optimizes the energy conversion in solar cells driven by conjugated polymer semiconductors is the electron–hole recombination process, i.e., exciton decay [9,10]. It is possible to manipulate some multifunctional donor–acceptor based conjugated materials so they viably provide the necessary charge carrier separation under solar irradiation [11]. These new intramolecular DA copolymer materials are used to absorb the sunlight of an appropriate frequency that can cover the whole wavelength spectrum and provide suitable electron charge mobility [12,13]. In light of this concept, continuous endeavors have been made toward discovering nanostructured conjugated DA systems that are able to utilize the fascinating photocatalytic diversity of organic and inorganic semiconductors under visible light illumination. Concomitantly, several varieties of photocatalyst-based semiconductors are widely utilized in the photocatalytic process and among them, polymeric carbon nitride (PCN) has been identified as an energized stable candidate for the corresponding evolution of hydrogen energy from water systems [14,15]. PCN is rapidly moving towards practical use due to its easy synthesis, abundance in Nature, low price, overall flexibility, high yielding manufacturing process, and its stability [16,17]. Numerous negative properties, such as a small surface area, high charge recombination rate, and small light absorption capability hinder further exploitation of the parental PCN and predict a negative photocatalytic performance [18,19]. Therefore, many scientists have explored a variety of fundamental approaches to modifying PCN networks, e.g., heterojunctions [20], noble metal deposition [21], doping [22,23], and morphological control [24,25], that have significantly improved its photocatalytic activity. Inspired by these developments, co-polymerization has come forth as a new prominent technique to accomplish this by using new energized organic conjugated co-monomers embedded within the framework of PCN to enhance its photocatalytic properties. The introduction of these organic motifs within the PCN structure via the process of co-polymerization optimizes the easy accumulation of the photogenerated electrons and accelerates their transport, hence fueling improved photocatalytic activity under visible light irradiation, as compared to the original PCN [26]. This co-polymerization grafts the given organic motifs within the skeleton of PCN using a facile one-step condensation process, and after the copolymerization of the PCN, the products demonstrate a high photocatalytic stability and performance. In light of this concept, Nie et al. created Z-scheme g-C_3_N_4_/ZnO microspheres for photocatalytic CO_2_ reduction [27,28]. Hayat et al. investigated use of a conjugated co-monomer, i.e., trimesic acid, within a PCN framework, which was successfully utilized for the photocatalytic CO_2_ reduction [29,30]. Intramolecular DA co-monomers are of great interest and are recognized as energized candidates that encroach within the triazine subunit of PCN to display their intramolecular donor–acceptor properties and enhance the photocatalytic performance of PCN [31,32].

**Scheme 1:**
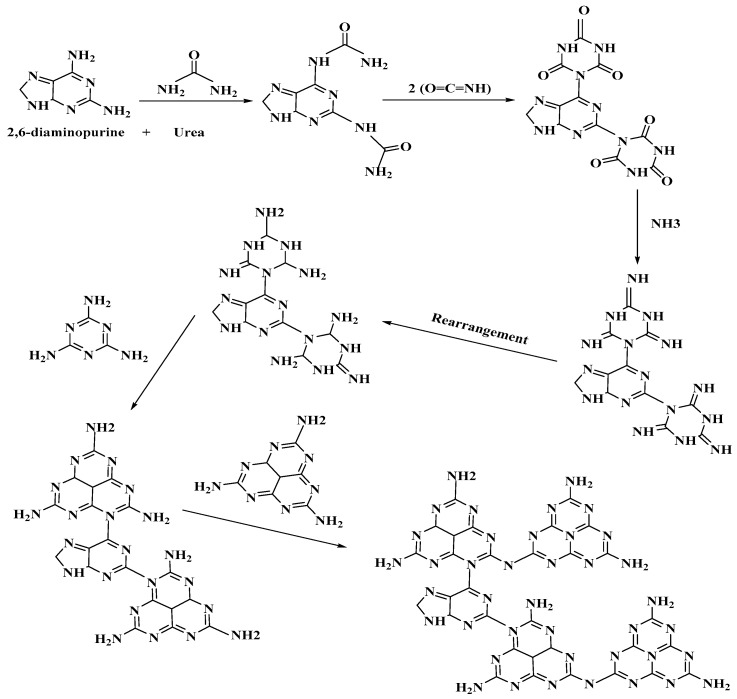
Possible reaction path for the integration of 2,6-diaminopurine into PCN networks.

This work utilized the aromatic electron–donor monomer 2,6-diaminopurine within the PCN framework to improve the photocatalytic reduction of CO_2_ into CO and H_2_ under visible light illumination. The installation of diamino aromatic moieties on the purine monomer was accomplished by effecting a nucleophilic substitution in the inner three s-triazine units of PCN thus building a great intramolecular donor-acceptor polymer for photocatalytic CO_2_ reduction as depicted in Scheme 1. More importantly, the monomer selected here forms heterocyclic pyrimidine and imidazole ring structures that correspondingly act as a backbone modifier [33,34]. The co-polymerization was repeated using appropriate amounts of different substances belonging to the same class of monomer produces such as 2,6-dihydroxypurine (xanthine), trimethylxanthine (caffeine) and methylxanthine (theobromine) in within the PCN skeleton to compare their photocatalytic CO_2_ reduction with the original samples. In short, we added different amounts of the electron donor-acceptor co-monomer 2,6-diaminopurine into the skeleton of urea-containing PCN by a facile one-pot thermo-induced co-condensation method and studied the products’ versatile application in photocatalytic CO_2_ reduction for the first time. It has been discovered that under visible light irradiation the as-synthesized samples exhibit an outstanding photocatalytic activity for the reduction of CO_2_ into CO and H_2_, about 16-fold up higher than that of the parental PCN. The density functional theory (DFT) calculations, different analytical techniques applied and the heterogeneous catalysis applications catalyzed by our samples described in this manuscript are expected to provide fruitful guidance for the fabrication of novel energetic polymeric photocatalysts.

## 2. Results and Discussion

**Figure 1:**
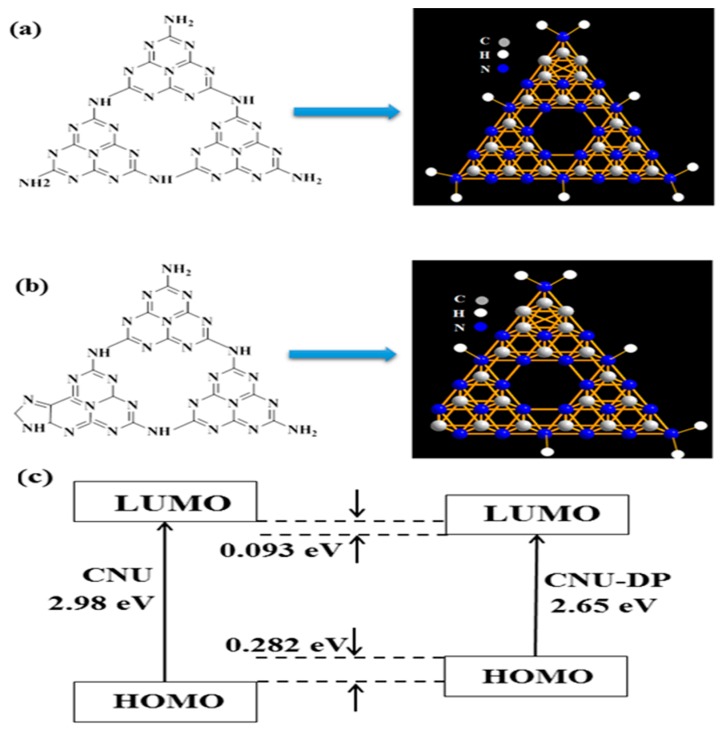
Electronic structure of polymeric trimer models including the optimized HOMO and LUMO for CNU trimer (**a**) and DP-doped CNU trimer (**b**), corresponding HOMO–LUMO gap (**c**).

The as-synthesized samples, i.e., CNU and co-polymerized CNU-DP_15.0_, were analyzed using DFT calculations to achieve a better visualization of their electronic structures. Along with this time-dependent DFT (TD-DFT) studies were conducted for the geometrical calculation of excited and ground states at the level of the Beck three-parameter hybrid functional (B3LYP)/6-31G (d, p) as implemented in Gaussian 09. As shown in Figure 1, the electronic distributions in the lowest unoccupied molecular orbital (LUMO) and highest occupied molecular orbital (HOMO) energy state of CNU and CNU-DP_15.0_ were elucidated (Figure S1). A high kinetic stability is indicated by the high HOMO-LUMO energy gap (ΔEg = 3.65 eV). Table S1 depicts the electronic excitation transition parameters of the CNU and CNU-DP_15.0_ samples and therefore the CNU-DP_15.0_ molecules possess high oscillator strength and dipole moment which emphasize their strong intermolecular electron push and full effect. The electronic excitation transition parameter ΔEST (S1-T1 = 0.39) change of the CNU-DP_15.0_ was also predicted using the B3LYP set and the results are summarized in Table S2. Table S3 lists the calculated values of Mulliken Atomic Charges (a.u.) for CNU-DP_15.0_ and it can be seen that the original sample attains a smaller band gap after the introduction of the monomer.

**Figure 2:**
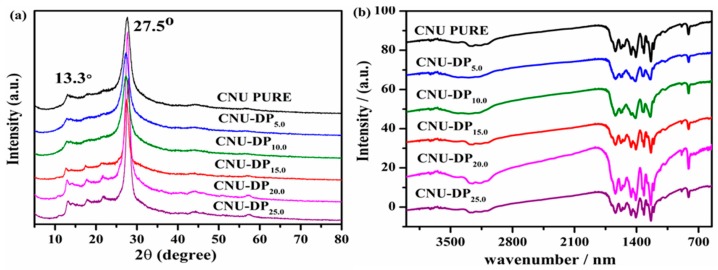
(**a**) XRD patterns and (**b**) FTIR spectra of CNU and CNU-DP_x_ samples.

The as-synthesized samples i.e., pristine CNU and co-polymerized CNU-DP_x_ samples were next subjected to X-ray diffraction pattern determination as depicted in Figure 2a. The final XRD characterization for all of the samples reflected no noticeable alterations and displayed the two main characteristic peaks at 27.5° and 13.1° assigned to the (002) and (100) planes of the crystal structure. The peak at 27.5° of the crystal structure face is a strong, broad and intense peak corresponding to the inter-planar stacking of aromatic systems [35,36]. All samples show these peaks and the aforementioned peak 27.5° peak due to disturbance of graphitic motifs resulting from the co-polymerization [37,38]. The peak displayed by all samples at 13.1° is a weak peak indexed to the (100) crystal plane which demonstrates the in-plane structural packing motif and the inter-planar stacking of aromatic systems [39,40]. It is noticeable that there is a small distortion in the XRD peak intensities in the range of ~15 to 25° that gradually increases with the increasing concentration of DP co-monomer in the CNU structure.

FT-IR spectra were recorded to verify the entire core chemical skeleton of PCN before and after the co-polymerization of DP co-monomer with the PCN. Multiple peaks were identified by analyzing the spectra illustrated in Figure 2b. The peaks located in the range between 2900 to 3300 cm^−1^ are broad peaks ascribed to the adsorbed H_2_O molecules and N-H vibrations originated from the partial condensation [41]. The peaks in the range between 1100–1800 cm^−1^ correspond to the typical stretching vibration modes of C-N and C=N heterocycles [42,43]. The peak appearing at 810 cm^−1^ originates from the significant bending vibration of the s-triazine motifs [44]. The characteristic FTIR peaks for the co-polymerized samples display a slight shift of the 810 cm^−1^ peak and therefore suggest a red shift due to the extra carbon content. The FTIR results reveal that all the samples display almost similar peaks and positions before and after the co-polymerization of DP co-monomer in the framework of PCN and hence no major alteration was revealed, hence confirming the successful deconvolution of the co-polymerization process.

**Figure 3:**
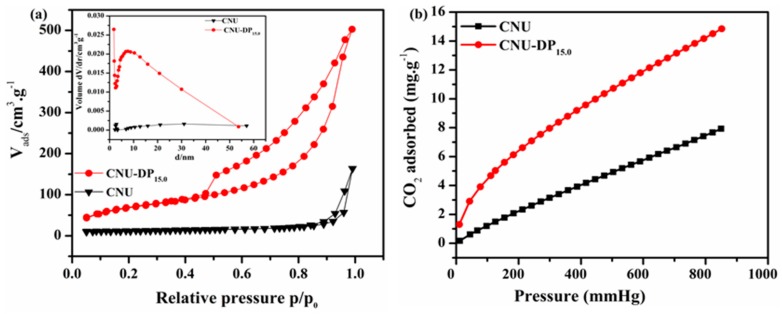
(**a**) N_2_ adsorption-desorption isotherms (77 K), (inset) pore size distribution graph, and (**b**) CO_2_ adsorption isotherms (273 K) for CNU and CNU-DP_15.0_ samples.

The pristine CNU and co-monomer polymerized CNU-DP_x_ samples were evaluated by N_2_ adsorption-desorption isotherms in order to detect their specific surface area, pore volume and aggregate porosity (Figure 3a). All the samples were subjected to sorption analysis using nitrogen gas as a sorbate and intrinsically a classic type IV isotherm was obtained, hence confirming the existence of mesoporous properties within all samples. After calculation, the pristine CNU sample gives a specific surface area of 39.89 m^2^g^−1^ and this surface area increases abruptly after the addtion of DCQ co-monomer in the CNU skeleton. Thereafter, the integration of various amounts of DP within the CNU sample result in an excellent surface area and thus the samples, i.e., CNU-DP_5.0_, CNU-DP_10.0_, CNU-DP_15.0_ and CNU-DP_20.0_ give surface areas of 131.98, 163.64, 214.53 and 202.2 m^2^g^−1^, respectively, as listed in Table S4.

More excitingly, the inset of Figure 3a presents the corresponding BJH pore size distribution of the samples and highlights a notable difference between the pure CNU and co-polymerized CNU-DP_x_ samples. The N_2_ adsorption-desorption isotherms and their corresponding BJH pore size distribution, pore size and volume of other co-polymerized samples are illustrated in Figure S2 and Table S4. The isotherms of simulated carbon dioxide adsorption of the parental CNU vs. CNU-DP_15.0_ were analyzed at 298 K and 1 bar atm. pressure, respectively. Here the simulated isotherms demonstrate a small overestimation due to some unremoved solvents. Interesting our CO_2_ adsorption isotherm indicates a type V behavior calculated at 273 K, as depicted in Figure 3b. Similarly the deducted isotherm showed a variable shape that was convoluted due to the pore size of samples and also manifested the electrostatic interactions of CO_2_ within the large-sized pores of the samples. The figure shows the CO_2_ adsorption isotherms as a comparison for the validation of the adsorption capabilities of CNU and CNU-DP_15.0_. In brief, the best CNU-DP_15.0_ sample displayed an extreme adsorption plot as compared of CNU and therefore manifests improved phenomena in its molecular sites which optimize the charge carrier probability during CO_2_ photofixation reactions.

**Figure 4:**
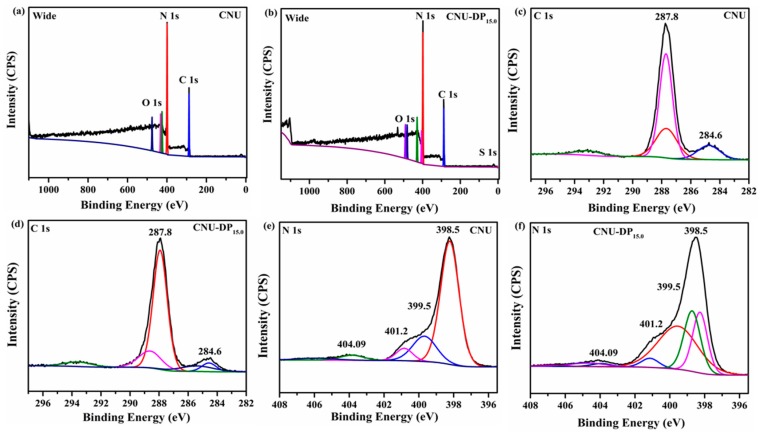
(**a**,**b**) XPS survey spectrum, (**c**,**d**) high-resolution XPS spectra of C 1s, and (**e**,**f**) N 1s for CNU and CNU-DP_15.0_.

XPS analysis was conducted to investigate the chemical status and elemental composition of the samples and also mirrored the effect before and after the co-polymerization of 2,6-diaminopurine within CNU. The XPS survey spectra demonstrate that C, N, O are present in all of the samples while a small amount of S was only detected in the co-polymerized CNU-DP_15.0_ sample as shown in Figure 4a,b. The high resolution XPS spectra of O assigned at 531 eV are presumed due to the presence of moisture in the atmosphere are depicted in Figure S3. The high resolution XPS spectra of S are interpreted as due to the incorporation of 2,6-diaminopurine co-monomer within the CNU as illustrated in Figure S4. Moreover the high resolution XPS spectra of C1 s for both CNU and CNU-DP_15.0_ show two distinct peaks and the apparent chemical shift in the binding energies (C, N) for both samples is depicted in Figure 4c,d. The peaks assigned at 284.6 ± 0.1 and 287.8 ± 0.1 eV for both samples are associated to the aromatic carbon atoms and the sp^2^ hybridized adventitious carbon (N-C=N and C-NH_2_) in the skeleton of PCN [45,46]. The high resolution N 1s XPS spectra for parental CNU and co-polymerized CNU-DP_15.0_ summarized in Figure 4e,f, demonstrated four peaks at different positions, i.e., at 398.5, 399.5, 401.2 and 404.09 eV, attributed to sp^2^-hybridized nitrogen from C-N=C bonds, the nitrogen within the tri s-triazine rings e.g., N-(C)_3_ group, terminal uncondensed C-N-H amino groups with implication of the charge diffused in the heterocyclic cage [47]. The atomic percentages of the elements derived from the XPS analysis are listed in Table S5. Meanwhile the elemental analysis of the samples was performed in order to confirm the carbon and nitrogen composition before and after the grafting of the aromatic 2,6-diaminopurine rings within the pristine CNU. The parental CNU manifested the calculated C/N molar ratio of 0.63 and this value successively increased after the addition of 2,6-diaminopurine co-monomer to the framework of PCN (Table S4). All of the co-polymerized samples display boosted C/N molar ratios and the best sample, i.e., CNU-DP_15.0_, capable of undergoing a more energized photocatalytic process, possesses a prodigious C/N molar ratio of 0.68.

**Figure 5:**
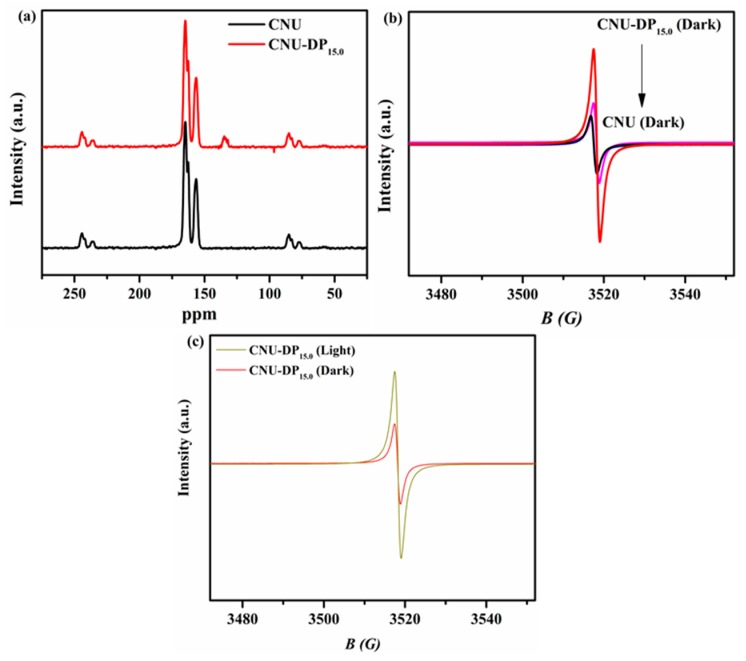
(**a**) Solid-state ^13^C-NMR spectra, (**b**) EPR spectra of CNU and different amount of CNU-DP_x_ samples, and (**c**) EPR spectra of CNU and CNU-DP_15.0_ in the dark and under visible light irradiation (>420 nm).

Solid-state ^13^C-NMR spectra were used to investigate the extra carbon added to the structure of CNU upon the co-polymerization of DP co-monomer. The solid-state ^13^C-NMR spectra manifested the expected differences between the spectra of the blank CNU sample versus co-polymerized CNU-DP_15.0_ as seen in Figure 5a. By optimizing the final resolution of the spectra, both of samples demonstrate similar obvious characteristic peaks at position 157.5 and 165.8 ppm associated to the 3-s-triazine-based PCN. A new weak peak appeared in the spectrum of CNU-DP_15.0_ photocatalyst as compared of CNU, demonstrating the incorporation of extra new carbon due to the copolymerization of DP co-monomer in the CNU framework. The electron paramagnetic resonance (EPR) spectra were recorded for all as-synthesized samples in order to mirror their calculated band structure as illustrated in Figure 5b. The result confirms that each catalyst has a single Lorentzian line tuning at position 3515G which increases during the co-polymerization process. The g-value of this single Lorentzian line is calculated as 2.0034 which is a feasible configuration due to delocalization of the π-conjugated system. Actually, the process of incorporation of co-monomer DP into the cage of CNU abruptly changes this π-conjugated system which results in an abundance of carbon that improves and regulate the photogenerated electron and hole-producing chain. This aforementioned process involves the association of unpaired electrons with the π-bonded nano-sized clusters on the CNU surface due to carbon atoms [48,49]. Figure 5c shows the EPR signals in both the dark and visible region of pristine CNU and the superior photocatalyst CNU-DP_15.0_. The final result leads to the conclusion that under visible light irradiation the signal intensity increases greatly, producing more photogenerated carriers.

**Figure 6:**
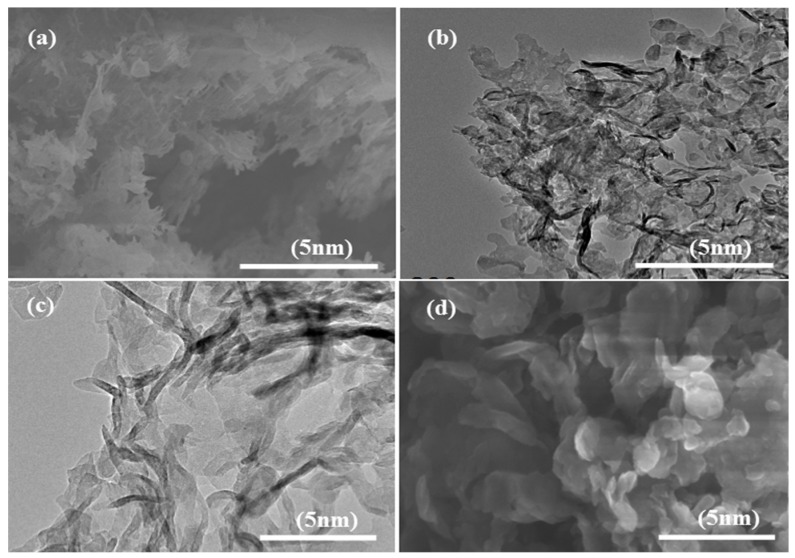
(**a**,**b**) the SEM images and (**c**,**d**) TEM images of CNU and CNU-DP_x_ samples.

The intra structure integrity of the as-synthesized samples was further studied by conducting typical SEM and TEM experiments as displayed in Figure 6. The morphology of pure CNU shows a distorted irregular lamellar scale which implies crystallinity (Figure 6a) while this morphology is converted into stacked agglomerated nanosheets of a few nanometers in size after the incorporation of DP co-monomer in the skeleton of CNU samples, as manifested in Figure 6b. The TEM image of pure CNU demonstrates a redundant isolated sheet which results in a diffused contrast level as displayed in Figure 6c. The TEM morphology of the co-polymerized CNU-DP_15.0_ sample reflects a dense sheet in the form of embedded belts which clearly show a strong degree of stacking as shown in Figure 6d.

**Figure 7:**
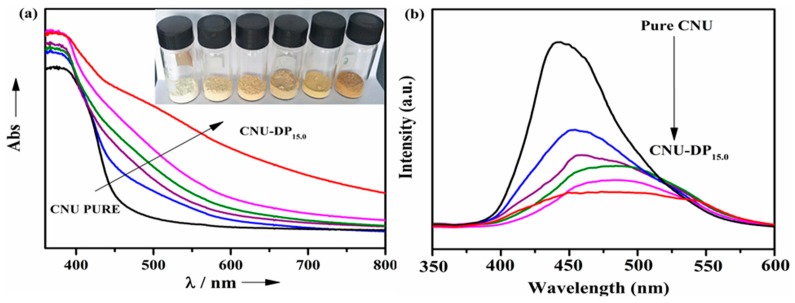
(**a**) UV–Vis DRS spectra of the CNU and CNU-DP_x_ samples and (**b**) the photoluminescence spectrum under 400 nm excitation.

Approaches such as UV–Vis DRS and photoluminescence (PL) spectroscopy were implemented for the exploration of the physicochemical properties of pure CNU and the effect of 2,6-diamino- purine co-monomer addition the urea skeleton in CNU. As displayed in Figure 7a, UV–Vis DRS shows the absorption edge of pure CNU at ca. 450 nm confirming the intrinsic band structure configuration. The absorption edge of the corresponding co-polymerized product, i.e., CNU-DP_x_ shows an enhanced net wavelength phenomenon as compared of pristine CNU. The UV–Vis DRS spectra are also reflected by macroscopic color changes from pale yellow toward a deeper brown hence confirming the enhancement of the visible light harvesting tendency of the inter-triazine oligomers of CNU by the successful process of co-polymerization, as seen in the inset of Figure 7a. More excitingly, the absorption optical edge of CNU was successfully red shifted after the addition of 2,6-diaminopurine co-monomer.

This process of co-polymerization of DP monomer within CNU therefore optimizes the consequent transition of photogenerated electrons that move from the HOMO toward the LUMO driven by the addition of carbon species. The average band gap of co-polymerized samples tends to decrease compared with the parent CNU as a function of their optical absorption, as manifested by DRS and also deduced from the DFT calculations. Figure 7b shows the PL spectra of pure CNU over co-polymerized CNU-DP_x_ samples in order to depict the excitation and separation of photo-generated electrons and holes. The samples demonstrate fluorescence emission decay around 445 nm and therefore a noticeable broad change in the PL emission intensity occurs in the co-polymerized CNU-DP_x_ samples. The ascribed alteration changes the PL emission toward longer wavelengths which justifies the existence of a charge carrier recombination process that is enhanced due to addition of DP co-monomer within the CNU framework by the supramolecular aggregation process. The enhancement in optical absorption hence lowers the charge recombination intensity and finally improves the electron photogeneration rate and tends to increase the transfer process from the valence band toward the conduction band rendering CNU-DP_x_ a useful material for photocatalytic reactions.

### 2.1. Photocatalytic CO_2_ Reduction

**Figure 8:**
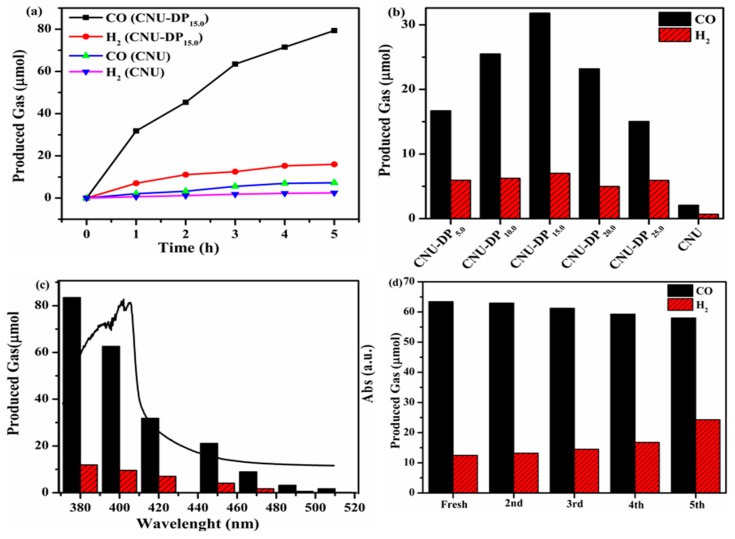
(**a**) Time-production plot of CO and H_2_ by photoreduction of CO_2_, using CNU-DP_15.0_ as a catalyst under visible light irradiation. (**b**) The effect of integration of different amounts of DPx into the CNU framework for CO_2_ photoreduction as compared to blank CNU. (**c**) The dependence of CO_2_ photo-reduction on different incident light wavelengths. (**d**) Stability of the CNU-DP_15.0_ as a photocatalyst.

The as-prepared samples i.e., parental CNU and co-polymerized CNU-DPx were utilized for CO_2_ reduction reactions under visible light irradiation. The reaction system was composed of a mixture of distilled water and the organic solvent acetonitrile (MeCN), Co(bpy)_3_^2+^ co-catalyst and TEOA which act a sacrificial electron donor. The organic MeCN is used as main solvent due to its great solubilization of the whole reaction system [50]. Figure 8 shows the evolution if the two main products, i.e., CO and H_2_, after the photocatalytic CO_2_ reduction reaction. The reaction system was subjected to photocatalysis during a four-hour reaction cyclic, resulting in a highest yield of 79.32 µmol of CO and 16.01 μmol of H_2_, therefore demonstrating a remarkable capability of CNU-DP_15.0_ as a superior photocatalyst as compared of the parental CNU (Figure 8a). The above results depict a boosted photocatalytic CO_2_ reduction rate with respect to the co-catalyst Co^2+^and confirms that this enhancement is the result of the introduction of 2,6-diaminopurine co-monomer within the skeleton of CNU. As discussed in the experimental section, varied amounts of co-monomer were added within the CNU samples in order to examine the effect of monomer amount on photocatalytic activity. Initially the parental CNU was applied to photocatalytic CO_2_ reduction therefore demonstrating the baseline activity as presented in Figure 8b. This photocatalytic activity increases abruptly after the co-polymerization of DP co-monomer within the CNU framework and therefore CNU-DP_15.0_ shows a remarkable catalytic activity with a CO evolution rate of 31.1 µmolh^−1^, almost 15.02 times higher than the parental CNU. This enhancement in the catalytic activity of CNU-DP_15.0_ depends on the co-polymerization process of light harvesting phenomena which is an important factor for accelerating the photoredox performance of PCN. It also demonstrates that this activity tends to decline when the amount of co-monomer increases beyond 15 mg. By integrating a large amount of co-monomer the conjugated shape of PCN is destroyed. The spinal photocatalyst CNU-DP_15.0_ was investigated predominantly for the evolution of CO/H_2_ products in term of changes of its optical absorption phenomena as depicted in Figure 8c. Before these wavelength experiments were optimized, a Xe lamp was employed by changing its filter cut-off in the visible range region and irradiating the concerned samples. The results indicate an outstanding evolution of CO and H_2_ products by modulating the intensity of the visible light and even display an energetically favorable photocatalytic performance at 510 nm as compared of pristine CNU. The above result verifies that the association of sunlight harvested photons efficiently accelerates the photochemical reaction of CO_2_ reduction into its main products. The superior CNU-DP_15.0_ sample was subjected to prolonged repetitive experiments in order to determine its stability for CO_2_ photo-reduction under visible light. The sample after use was collected, washed, filtered and added into a fresh reaction and investigated for five cycles. As seen from Figure 8d, the product is eminently stable and therefore no any change was observed in the evolution of CO/H_2_ products. The vitality of the stability protocol depends on the activation of the co-catalyst cobalt species and hence there a rejuvenated optimized production of CO/H_2_ showing a TON of ca. 170, which is a notable achievement in photocatalytic diversity. After prolonging the experiments, the sample was recollected by washing and utilized for structural characterizations such as XRD, FTIR, DRS and XPS for comparison with fresh a sample as depicted in Figures S5 and S6. As suggested from the above results that was no noticeable change in the crystalline, chemical, and surface structure of the used samples, therefore indicating a highly stable photocatalyst for the photoreduction of CO_2_.

**Figure 9:**
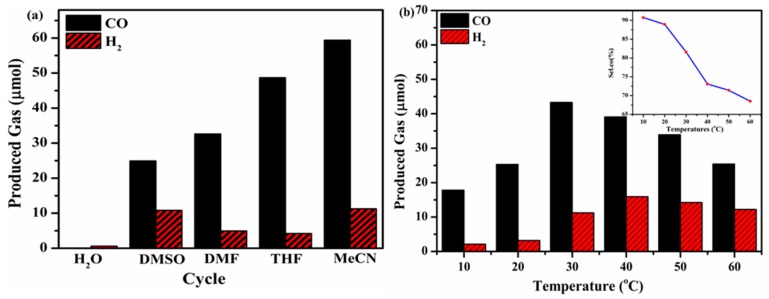
(**a**) Various control experiments of CNU-DP_15.0_ photocatalyst using different organic solvents for the generation of CO and H_2_ under visible light. (**b**) Evolution of CO and H_2_ at various reaction temperatures (inset shows the selectivity).

To confirm that the photocatalytic CO_2_ reduction reaction was really dependent on the effect of the reaction solvent, several control experiments were performed utilizing different protic solvents (e.g., MeCN, DMSO, THF, DMF and H_2_O) as illustrated in Figure 9a. The results demonstrate that when pure H_2_O was used in the reaction system, no detection of CO or H_2_ products occurred. When organic solvents were utilized individually in each reaction system, i.e., MeCN, DMSO, THF, or DMF a number of potential products were extracted. Among the aforementioned solvents, acetonitrile (MeCN) displayed exclusive photocatalytic CO_2_ reduction activity under visible light irradiation. The above result manifests no interaction between the CO_2_ molecules and desired solvent and explains the optimized CO_2_ reduction process [33]. Several protocols have been optimized by utilizing CNU-DP_15.0_ photocatalyst for the photoreduction reaction of different CO_2_ sources and therefore several reactions were addressed in different temperature regions (10–50 °C) in order to establish the dependence of the evolution of CO on the reaction temperature. As depicted in Figure 9b, the evolution of CO showed an enhanced yield of 45.37 μmol/h at 30 ^o^C with an excellent selectivity of 81.58 % as seen in the inset of Figure 9b. Below and above this temperature, the CO_2_-to-CO conversion rate slightly declined, therefore confirming 30 ^o^C as the optimal reaction temperature for this chemical system. At high temperature an abundant amount of CO_2_ molecules is discharged from the reaction system which has a negative influence on the economical catalytic performance of CO_2_ reduction.

**Figure 10:**
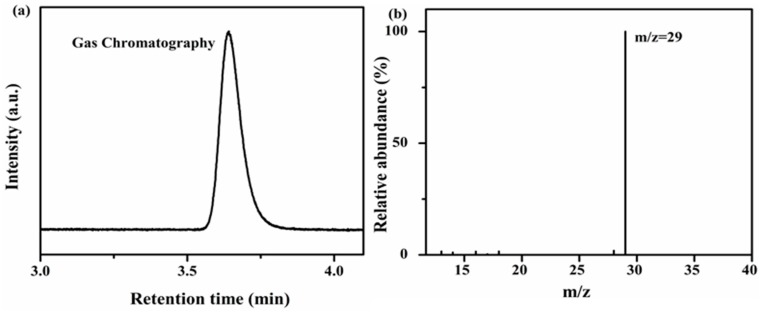
(**a**) Mass spectra (*m*/*z* = 29), and (**b**) gas chromatogram of the carbon source of the evolved CO in the photocatalytic reduction of ^13^CO_2_.

Isotopic experiments have been performed using ^13^CO_2_ gas under the same reaction conditions in order to investigate the source of the evolved CO product. The CO photocatalytically evolved after two hours of irradiation was analyzed by gas chromatography-mass spectrometry (GC-MS). The net result of the GC chromatogram was a peak at 3.87 min having *m*/*z* = 29 therefore attributed to ^13^CO as presented in Figure 10a,b. The aforementioned results prove that during the reaction, the evolved CO is indeed produced due to a reductive deoxygenation of the CO_2_ reactant under visible light under mild conditions. The net photocatalytic performance of CO_2_ reduction was intrinsically moderate, which was intimately affected by the consistent stability of CO_2_ molecules with their alternating configuration [51,52]. The photocatalysis of CO_2_ reduction combined with induced photons are more favored than direct CO_2_ reduction. For this, we replaced the water solvent with MeCN to optimize the reaction medium, and further accomplished the tuning between the photocatalytic activity and the volumetric H_2_O gradient. As depicted in Figure S7a, when water was introduced into the reaction system along with acetonitrile then the photocatalytic activity of the CO_2_-to-CO conversion was intensively accelerated when the best CNU-DP_15.0_ sample was utilized. In control experiments, we studied different water/acetonitrile ratios (0/6, 1/5, 2/4, 3/3, 4/2) and ultimately the 1/5 volumetric ratio showed a remarkable catalytic activity under the applied conditions, which is about five time higher than that of the non-water-added system. This promotion in the photocatalytic activity is attributed to the water content in the reaction system which lowers the thermodynamic barrier and accelerates the reaction kinetics. The addition of water also improved the reaction with a Ru-photosensitizer which generates the active species for the photocatalytic reaction [53]. When the content of water was increased more an inhibitory effect occurred, which caused a decline in the photocatalytic CO_2_ reduction reaction. Figure S7b demonstrates the CO_2_ photo-reduction by utilizing monomers similar of 2,6-diaminopurine but different group attachments and compares its evolution products with the best sample, also illustrated in Table S6. Several control experiments were conducted in order to determine various parameters that impact the reaction system are illustrated in Table S7. Initially, the reaction was conducted without the inclusion of either CNU or light, resulting in no evolution of CO and H_2_ (Table S7). The result reflected that the cobalt ion co-catalyst part without the inclusion of the main catalyst cannot drive the CO_2_ reduction reaction. The presence of semi-conductor photocatalyst along with cobalt ions absorbs a high amount of light to produce photogenerated electrons and facilitate their transport from the valence band toward the conduction band resulting in the conversion of the CO_2_ source. Some control experiments were performed without the induction of sacrificial agent and co-catalyst therefore demonstrating a negligible amount of CO products, as shown in Table S7. The CO_2_ gas was replaced instead with nitrogen and argon gas in the reaction system and hence no evolution of CO products occurred, therefore demonstrating the degradation influence of the main catalyst, i.e., PCN, along with other organic compounds such as TEOA and bpy. Some reference reactions depicted that the photocatalytic CO_2_ reduction terminated without the inclusion of cobalt ions or ligands or both (Table S7). The basic role these ligands is to act as electron mediators during the photoreductive process, further analyzed by the PL spectra of the system as discussed before.

### 2.2. Photoelectrochemical Measurements

**Figure 11:**
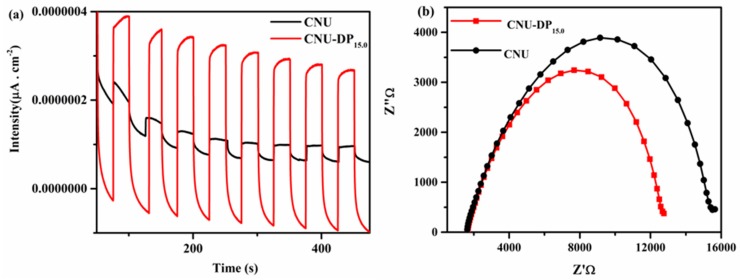
(**a**) Periodic on/off photocurrent (**b**) the EIS Nyquist plots b/w CNU & CNU-DP_15.0_ at Ag/AgCl in a 0.2M Na_2_SO_4_ aqueous solution (pH 6.8) response under visible light irradiation (λ=420 nm).

**Figure 12:**
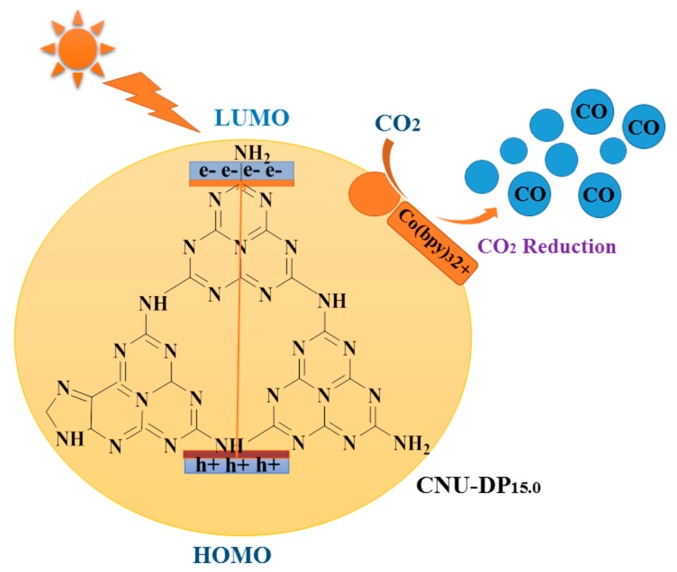
Schematic proposed mechanism of photocatalytic CO_2_ reduction.

As mentioned before the photocatalytic activity of co-polymerized CNU-DP_15.0_ displayed an optimized efficiency in terms of CO_2_ reduction as compared to blank CNU samples, which can be mainly associated to the constitutional charge mobility of photogenerated electrons and holes in the co-polymerized samples. Photoelectrochemical measurements have been performed for both the parental CNU and co-polymerized CNU-DP_15.0_ samples in order to establish the apparent tendency of charge carriers and their transport process within these samples. Figure 11a shows the transient photocurrent density curves of blank CNU and co-polymerized CNU-DP_15.0_ samples and therefore manifests a drastic cathodic photocurrent spike for the specific on/off light interval under solar irradiation (λ = 420 nm). The photocurrent density course of the as-polymerized CNU-DP_15.0_ sample is extremely enhanced compared to that of the parental CNU, suggesting that the conjugated electron donor-acceptor monomer interaction within the framework of CNU hence ameliorates the efficiency of the charge transfer process. Meanwhile, electrochemical impedance spectroscopy (EIS) measurements were evaluated for both of samples in the dark region in order to verify the above conclusion, as depicted in Figure 11b. The result demonstrates the charge transfer resistances of pure CNU and co-polymerized CNU-DP_15.0_ are 324.9, and 277.1 Ώ, respectively. The decrease in semicircular Nyquist plots (resistance capacity) of the co-polymerized CNU-DP_15.0_ as compared to CNU suggests a superior reaction rate on the surface of the CNU-DP_15.0_ electrode. This promotion in the reaction mechanism of the co-polymerized sample manifests as an energized transport and separation of photogenerated charge carriers at the surface. It is of great importance that the integration of a high amount of purine within the CNU structure hence decreases the LUMO energy state of the acceptor which intimately results in a decline in the photocatalytic activity of CO_2_ reduction. This incorporation of a high amount hence effects a destruction of the structure of PCN and therefore an extensive amount of photogenerated electrons will be emitted in the form of vibrational/thermal energy, etc. which is also a key factor to lower the photocatalytic activity of CO_2_ reduction. The above conclusion postulates the assumption that the integration of conjugated donor-acceptor DP monomer within the matrix of CNU significantly enhances the photogenerated electrons and holes and also accelerates its transportation strikingly. The detailed photocatalytic mechanism is as shown in Figure 12. Due to this co-polymerization, the resultant surface phenomena and optical absorbance rate of PCN was remarkably increased, which results in a lowered rate of electron-hole recombination making it a superior material for heterogeneous photocatalytic CO_2_ reductions.

## 3. Experimental Section

### 3.1. Materials

All chemicals used in synthesis and photocatalytic reaction were utilized directly without any further purification. In all the experiments the distilled water supplied by an appropriate internal laboratory apparatus is mandatory. Urea (>99%) was supplied by Sinopharm Chemical Reagent Co. Ltd. (Fuzhou, China) and 2,6-diaminopurine, trimethylxanthine (caffeine) and methylxanthine (theobromine) 2,2-bipyridine (bpy), CoCl_2_, H_2_PtCl_6_ were obtained from Sigma Aldrich (Fuzhou, China). The organic solvents such as triethanolamine (TEOA), trichloromethane (TCM), N,N-dimethylformamide (DMF), acetonitrile (MeCN) and tetrahydrofuran (THF) were bought from Sinopharm Chemical Reagent Co., Ltd.. The main gases such as carbon dioxide (CO_2_) and ^13^CO_2_ were provided from Fuzhou Lianzhong Industrial Gases and Hess Chemical gas Center (Beijing, China).

### 3.2. Synthetic Procedures

In a typical procedure, PCN was synthesized by taking 10 g of urea in 15 mL of distilled water and heating at 80 °C for a day and a night in an oil bath system under continuous stirring in order to evaporate the water completely. After, the resultant products were transferred into an alumina crucible with a cover and heated to 600 °C for 2 h at a heating rate of 5 °C/min. After performing various polycondensation processes at the appropriate temperature, the as-synthesized samples were collected, ground and named CNU. The co-polymerized samples were prepared by taking 10 g of urea with different amount of co-monomer, i.e., 2,6-diaminopurine in 15 mL of distilled water and keeping the mixture in an oil bath at 80 °C for a day and a night until a solid residue was left. The solid products were transferred to an alumina crucible with a cover and heated to 600 °C for 2 h at a heating rate of 5 °C/min and named CNU-DP_x_ where x is the different amount of given monomer and is equal to 5, 10, 15, 20 and 25 mg. For comparison, the same class of monomer but with different groups such as 2,6-dihydroxypurine (xanthine), trimethylxanthine (caffeine) and methylxanthine (theobromine) were also introduced within the PCN framework giving species such as CNU-XT_15.0_, CNU-CF_15.0_ and CNU-TB_15.0_ using the above procedure. The amount of co-monomer embedded in other precursors of PCN was kept as 15 mg because this monomer amount in the CNU showed superior activity.

### 3.3. Characterization

The as-prepared samples were examined by X-ray diffraction (XRD) measurements on a Bruker D8 Advance diffractometer (Bruker, Germany). A Nicolet Magna 670 Fourier transform infrared spectrometer (Thermo Electron Corp., Madison, WI, USA) was used to record the Fourier transform infrared (FT-IR) spectra of all samples. The X-ray photoelectron spectroscopy (XPS) measurements were performed on an ESCALAB250 instrument (Thermo, Thermo, VG Microtech, UK) equipped with a monochromatized Al Ka line source (200 W). The samples were subjected to solid-state ^13^C-NMR experiments that carried out on a Bruker Advance III 500 spectrometer. The electron paramagnetic resonance (EPR) measurements were conducted on a Bruker model A300 spectrometer. The emission scanning electron microscopy (FESEM) and transmission electron microscopy (TEM) experiments were performed a New Generation SU8010 (Hitachi, Japan) and a model JEM 2010 EX (JEOL, Tokyo, Japan) instrument, respectively. The UV–Vis diffuse reflectance spectra (DRS) of as-synthesized samples were run on a Cary 500 Scan UV–Vis spectrophotometer (Varian, Darmstadt, Germany) with barium sulfate as the reference. A FI/FSTCSPC 920 spectrophotometer (Edinburgh, UK) was used for the measurement of the photoluminescence (PL) spectra of all samples. The N_2_ adsorption-desorption and CO_2_ adsorption isotherms were characterized on an ASAP2020 system (Micromeritics, Boston, MA, USA). The photocatalytic produced gases were analyzed by an 7820A gas chromatograph (Agilent, Boston, MA, USA) equipped with a thermal conductivity detector (TCD) and a TD-01 packed column. The ^13^CO_2_ isotopic experiment for the detection of ^13^CO gas was performed on a HP 5973 gas chromatography-mass spectrometry (GC–MS) system.

### 3.4. Photocatalytic CO_2_ Reduction Test

The photocatalytic CO_2_ reduction experiments were performed under visible light irradiation by utilizing a 300 W Xe lamp with a 420 nm cut-off filter. The reaction was done in an 80 mL Schlenk flask reactor that contained 30 mg of photocatalyst, 24 mg CoCl_2_·6H_2_O, 15 mg 2,2-bipyridine, 1 mL triethanolamine (TEOA) dissolved in 6 mL of solvent, i.e., water/acetonitrile (H_2_O/MeCN = 1 mL/5 mL). Before irradiation, the reaction system was degassed and backfilled under vigorous stirring with pure CO_2_ gas by a connected air pump and finally pure CO_2_ (1 atm) was added to the reaction system. The temperature of the reaction system was controlled at 30 °C with a water cooling system and set up for irradiation under visible light and stirring. After irradiation, the produced gases were detected by the Agilent 7820A gas chromatograph. ^13^CO_2_ gas was utilized as reactant for conducting the isotopic experiments under the same photocatalysis conditions and after irradiation the products were analyzed using gas chromatography-mass spectrometry (GC–MS).

### 3.5. Electrochemical Analysis

The electrochemical measurements used working electrodes which were pretreated by the doctor-blade method. The paste was coated on a fluorine-doped tin oxide (FTO) substrate cleaned and washed several times with ethanol, acetone and distilled water, respectively. The as-synthesized samples i.e., parental CNU and CNU-DP_15.0_ (5 mg) were used to make fine slurries by dissolving them in 2 mL of dimethylformamide (DMF) under ultrasonication. The as-prepared slurries were pasted onto the FTO electrodes after drying in air for a day and a night to obtain CNU and CNU-DP_15.0_ photocatalysts. The electrochemical analyses were conducted by using a CHI electrochemical workstation (BioLogic VSP-300, Paris, France) that is composed of three conventional electrodes, i.e., working, reference and counter electrode. An electrolyte of 0.2 M sodium sulfate electrolyte solution (Na_2_SO_4_) aqueous solution were used in which the working electrode i.e., CNU and CNU-DP_15.0_, counter electrode Pt sheet and the reference electrode Ag/AgCl were immersed. A Xe lamp was used as irradiation source that illuminate the working electrode from the back side (FTO substrate/semiconductor interface) in order to decrease the effect of semi-conductor thickness. All parts of the working electrode were covered, and only 0.2 cm^2^ areas are exposed for light illumination. The photocurrent measurement such as periodic on/off response of CNU and CNU-DP_15.0_ electrode is indexed at 0.4 V bias vs. Ag/AgCl and the perturbation signal was applied at 10 mV with a frequency of 1 kHz. During the electrochemical impedance spectroscopy (EIS) experiments, the perturbation signal was similarly 10 mV, obtained by alternating the frequency range from 200 kHz to 10 mHz.

## 4. Conclusions

Numerous proposed intramolecular conjugated monomers are quenched by the co-polymerization process or are unusable due to their complex synthesis or high cost. Conjugated donor-acceptor co-polymers are considered an efficient rejuvenated candidate due to their superior performance, low cost, abundance and a stabilized organic conjugated co-monomer was introduced into the main skeleton of PCN to enhance to photocatalytic activity. The optimization of the conjugated donor-acceptor 2,6-diaminopurine co-monomer copolymerized with the host PCN has been postulated. This conjugated donor-acceptor nitrogen-rich co-monomer DP is essentially composed of imidazole rings grafted by a pyrimidine (electron-donor/electron acceptor) that are considered a superior donor-acceptor system and energetically stable. This integration of DP co-monomer with PCN semi-conductor accelerates a nucleophilic reaction at the position of the adjacent amino group due drive supramolecular aggregation (co-polymerization) and form a long polymer having high surface area and better photocatalytic activity than the parent compound. The as-fabricated samples were successfully analyzed using different characterization techniques to explain its electronic, optical properties, crystal phase, microstructure and morphology that were influenced by the interaction with the DP co-monomer. DFT calculations on pure PCN and the CNU-DP_15.0_ material trimers (interpreted as the result of incorporation of the co-monomer at two different positions) were comprehensively evaluated and they exhibited a remarkable structural optimization without the inclusion of any symmetry constraints and also allows manipulation of their optical and electronic properties. The incorporation of the donor-acceptor (D-A) 2,6-diaminopurine co-monomer within PCN influences the chemical affinities, polarities and acid–base functions of PCN and enhances the photocatalytic activity for CO_2_ reduction to CO and H_2_ and also improves its selectivity. The present work presents an interesting concept and validates the experimental and theoretical information for developing a catalyst with enhanced photocatalytic performance.

## Supplementary Materials

The supplementary materials are available online. Figure S1: Calculated spatial electron distributions of HOMO and LUMO for CNU and co-polymerized CNU-DP_15.0_. Figure S2: N_2_ adsorption-desorption isotherms (77 K), (inset) pore size distribution graph, for (a) CNU and CNU-DP_10.0_ and (b) CNU and CNU-DP_20.0_samples, respectively. Figure S3: XPS results of high-resolution spectra of O 1s for CNU and CNU-DP_15.0_. Figure S4: XPS results of high-resolution spectra of S 1s for CNU and CNU-DP_15.0_. Figure S5: XRD (a) and FTIR (b) for fresh sample and used sample of CNU-DP_15.0_. Figure S6: C 1s (a) and N 1s (b) UV-Vis DRS (c) for fresh sample and used sample of CNU-DP_15.0_. Figure S7: (a) Utiilizing CNU-DP_15.0_ photocatalyst by using different volumetric ratio of water and acetonitrile (b) same class of monomer having different groups for the generation of CO and H_2_ under visible light. Table S1: Electronic excitation transition parameters of CNU and Co-polymerized CNU-DP_15.0_. Table S2: Electronic excitation transition parameters of CNU-DP_15.0_. Table S3: Calculated values of Mulliken Atomic Charges (a. u.) for CNU-DP_15.0_, DFT/ B3LYP/6-31G method. Table S4: Physicochemical properties of COand H_2_ evolution during CO_2_ reduction of as-prepared samples. Table S5: The element derived by XPS results. Table S6: Comparison of as-prepared samples with same class of co-monomers having different groups. Table S7: Study of various conditions on controlled experiments.
