# Supplementary material for: Conjugated Electron Donor–Acceptor Hybrid Polymeric Carbon Nitride as a Photocatalyst for CO2 Reduction
Source: Molecules. 2019 May 8;24(9):1779. doi: 10.3390/molecules24091779 (PMC6539331; doi:10.3390/molecules24091779)
Supplement: Supplementary file 1 [file molecules-24-01779-s001.pdf]

Supporting information

Conjugated Electron Donor–Acceptor Hybrid Polymeric Carbon Nitride as a Photocatalyst for CO_2 Reduction

Asif Hayat [2], Mati Ur Rahman [2], Iltaf Khan [3], Javid Khan [4], Muhammad Sohail [5], Humaira Yasmeen [6], Shu-yuan Liu [7,8,*], Kezhen Qi [1,*] and Wenxiu Lv [1]

[1] Institute of Catalysis for Energy and Environment, College of Chemistry and Chemical Engineering, Shenyang Normal University, Shenyang 110034, China; 15164052089@163.com
[2] College of Chemistry, Fuzhou University, Fuzhou 350002, China; asifncp11@yahoo.com (A.H.); matiurrahman617@yahoo.com (M.U.R.)
[3] Key Laboratory of Functional Inorganic Material Chemistry, School of Chemistry and Materials Science, Heilongjiang University, Harbin 158308, China; iltafkhanpakistan@gmail.com
[4] MOE Key Laboratory of Bioinorganic and Synthetic Chemistry, Key Laboratory of Environment and Energy Chemistry of Guangdong Higher Education Institutes, School of Chemistry, Sun Yat-Sen University, Guangzhou 510275, China; javidchemist@yahoo.com
[5] Institute for Advanced Study, Shenzhen University, Shenzhen 518060, China; sohailncp@gmail.com
[6] Key Laboratory of Bio-Based Material Science and Technology, Ministry of Education, Northeast Forestry University, Harbin 150040, China; humairanefu@yahoo.com
[7] Department of pharmacology, Shenyang Medical College, Shenyang 110034, China
[8] Key Laboratory for Photonic and Electronic Bandgap Materials, Ministry of Education, School of Physics and Electronic Engineering, Harbin Normal University, Harbin 150025, China
* Correspondence: liushuyuan@symc.edu.cn (S.-y.L.); qkzh2003@aliyun.com (K.Q.)

Academic Editors: Alireza Khataee, Mahdie Safarpour and Sang Woo Joo
Received: 18 April 2019; Accepted: date; Published: date

Density Functional Theory analysis

1.1. Materials and methods

The Gaussian 09 **[1]** software at the B3LYP/6-31G* level and time-dependent density functional theory (TD-DFT) method were used for quantum chemistry calculation. The (CNU-$DP_{15.0}$) molecule ground state geometries (S_0) were determinde from its single crystle structure and the further n_0 geomatery escalition was performed.

1.2. Time-dependent density functional theory (TD-DFT) calculations

The TD-DFT calculation was conducted on Gaussian 09 program according to the previous reported literature previous literature **[1]**. In order to maintain the corresponding intermolecular locations and their specific molecular configurations, the (S_0) geometries of the CX crystal were achieved from its single crystal structure CX11 and further n_0 geomatery escalition was performed. The geometry optimization based on B3LYP/6-31G (d)[2] was used to obtained the ground state (S_0) geometries of free CX molecule in vacuum, along with this the combination of TD-B3LYP/6-31G (d)[3] give the corresponding ground state geometries the n-th triplet (T_n) and n-th singlet (S_n) states **[3]**.The possible singlet-triplet intersystem crossings (ISC) mechanism was elucidated from Kohn-Sham frontier orbital analysis**[2]**.The major ISC channels are ascribs: the ergy gap of the same transition configuration in S_1 and T_n states should be small while the ratio in all tansition orbital copmosition should be larger.When the energy of S_1 is hogher than T_n, the first element will be considered as predominant. The minor ISC channels findings is vice versa.

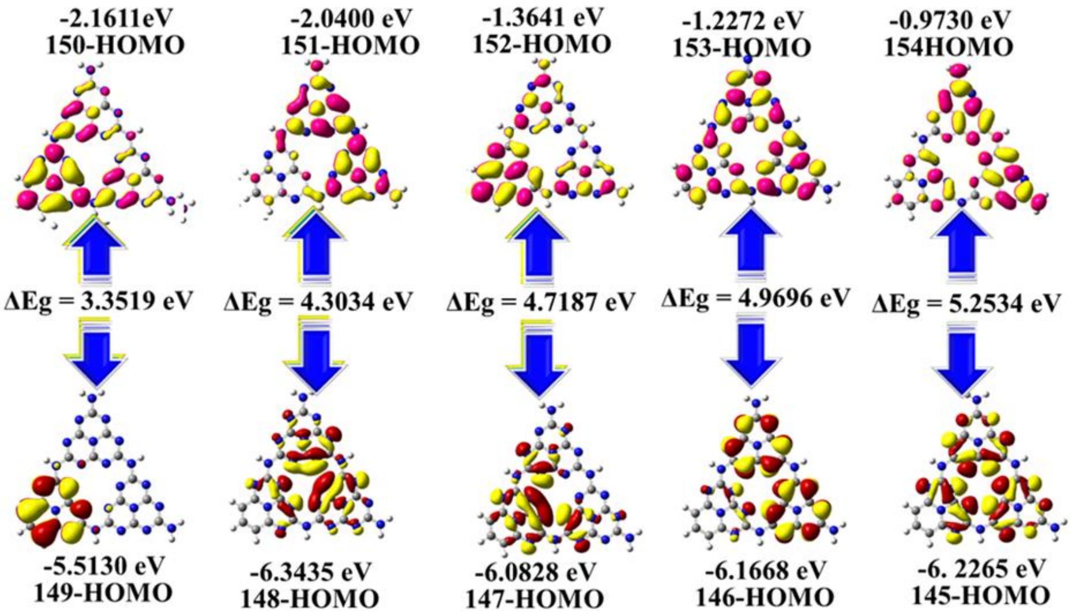

Figure S1. Calculated spatial electron distributions of HOMO and LUMO for CNU and co-polymerized CNU-DP$_{15.0}$.

Table S1: Electronic excitation transition parameters of CNU and Co-polymerized CNU-DP$_{15.0}$.

**Energy level**				**ΔE$_{ST}$ (eV)**	**Oscillator strengths**	**Dipole moment**
**Triplet (eV)**		**Singlet (eV)**				
**CNU sample**						
T1	-1.2159	S1	0.3602	1.2871	0.0005	3.6239 D
T2	0.4894	S2	0.295	0.2386	0.0006	
T3	0.5315	S3	1.8004	0.7629	0.0057	
T4	0.8139	S4	1.1285	0.1526	0.0098	
T5	1.2747	S5	1.2806	0.2189	0.0067	
T6	1.2592	S6	3.1756	1.4194	0.0047	
**CNU-DP$_{15.0}$ sample**						
T1	2.0587	S1	2.9174	0.3697	0.0032	2.1084 D
T2	2.2526	S2	2.8424	0.1928	0.0045	
T3	2.1189	S3	2.7057	0.2878	0.0174	
T4	2.4280	S4	2.9092	0.1892	0.0521	
T5	2.8358	S5	2.5228	0.174	0.0412	
T6	2.9894	S6	2.8609	0.1825	0.0005	

Table S2. Electronic excitation transition parameters of CNU−$DP_{15.0}$.

Energy level				ΔE_{ST} (eV)	oscillator strengths	dipole moment
triplet (eV)		singlet (eV)				
T1	2.0659	S1	3.9628	0.3019	0.0214	11.5279 D
T2	2.0516	S2	3.8792	0.4827	0.0123	
T3	3.1942	S3	3.2190	0.3879	0.0129	
T4	3.9891	S4	3.8584	0.3521	0.0241	
T5	3.8029	S5	3.9008	0.2753	0.0009	
T6	3.8562	S6	3.2575	0.1357	0.0007	

Table S3. Calculated values of Mulliken Atomic Charges (a. u.) for CNU−$DP_{15.0}$, DFT/ B3LYP/6-31G method**.**

Atoms	DFT	Atoms	DFT	Atoms	DFT
1 N	-0.614757	25 C	0.720706	49 C	0.002560
2 C	0.623481	26 N	-0.493512	50 N	0.226075
3 N	-0.196437	27 C	0.634777	51 N	-0.277632
4 C	0.587995	28 N	0.137083	52 N	-0.562150
5 N	-0.584606	29 C	0.627839	53 N	-0.595204
6 C	0.844876	30 N	-0.199201	54 N	-0.568197
7 N	0.203836	31 C	-0.598994	55 H	0.425361
8 C	0.822502	32 N	-0.240069	56 H	0.439019
9 N	-0.167459	33 N	-0.225642	57 H	0.281184
10 C	-0.405567	34 C	-0.174090	58 H	0.406083
11 N	-0.130080	35 N	-0.233759	59 H	0.404105
12 N	-0.208058	36 N	-0.828052	60 H	0.258313
13 C	-0.223623	37 N	-0.612051	61 H	0.225812
14 N	-0.180917	38 N	-0.177084	62 H	0.238946
15 N	-0.332764	39 C	0.737616	63 H	0.240013
16 N	-0.597430	40 N	-0.604536	64 H	0.393123
17 C	-2.345304	41 C	0.878909	65 H	0.381438
18 C	0.188745	42 N	-0.252502	66 H	0.398914
19 C	-1.698785	43 C	-0.229839	67 H	0.402286
20 C	0.292116	44 C	0.998575	68 H	0.308944
21 C	-0.974875	45 N	-0.278052	69 H	0.394509
22 C	0.441097	46 C	-0.196684	70 H	0.536620
23 C	0.335426	47 N	-0.250977	71 H	0.404067
24 N	-0.303845	48 N	-0.169212	72 H	0.401292

Table S4. Physicochemical properties of COand H_2 evolution during CO_2 reduction of as-prepared samples.

**Sample**	**Surface area[a] (m^2g^{-1})**	**Band gap (eV)[b]**	**Pore volume (cm^3 g^{-1})**	**Pore diameter (nm)**	**C/N molar ratio[c]**	**H_2[c] evolution (μmol h^{-1})**	**CO[d] evolution (μmol h^{-1})**
CNU	39.89	2.53	0.42	28.2	0.63	0.68	2.08
CNU−$DP_{5.0}$	131.98	2.25	0.46	21.4	0.66	5.93	16.7
CNU−$DP_{10.0}$	163.64	2.10	0.48	23.9	0.67	6.25	25.5
**CNU−$DP_{15.0}$**	**214.53**	**2.05**	**0.44**	**25.1**	**0.68**	**7.02**	**31.81**
CNU−$DP_{20.0}$	202.24	2.19	0.46	19.2	0.68	4.98	23.2
CNU−$DP_{25.0}$	137.06	2.21	0.44	20.8	0.67	5.92	15.05

[a]Calculated from N_2 absorption–desorption isotherms.

[b] From reference **[4]**

[c]From elemental analysis spectra.

[d] From CO_2reduction controlled experiments.

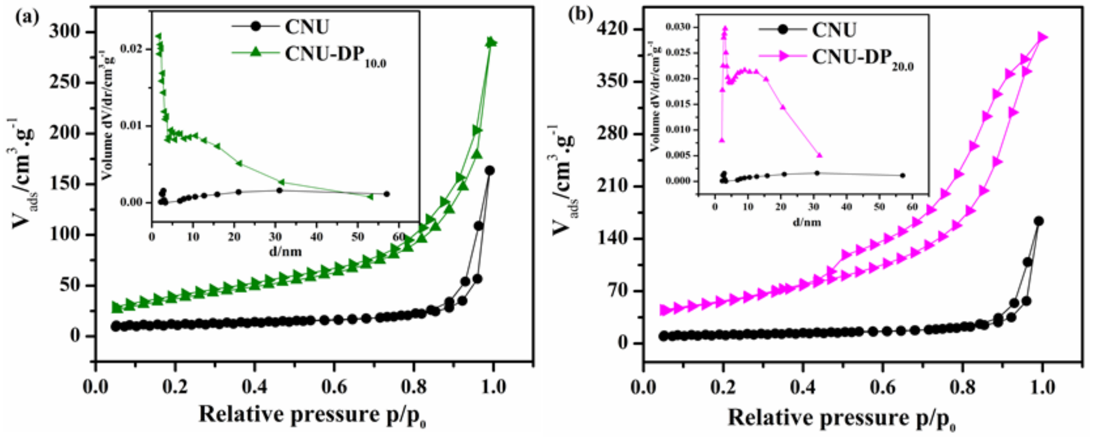

Figure S2. N_2 adsorption-desorption isotherms (77 K), **(inset)** pore size distribution graph, for **(a)** CNU and CNU–$DP_{10.0}$ and **(b)** CNU and CNU–$DP_{20.0}$samples, respectively.

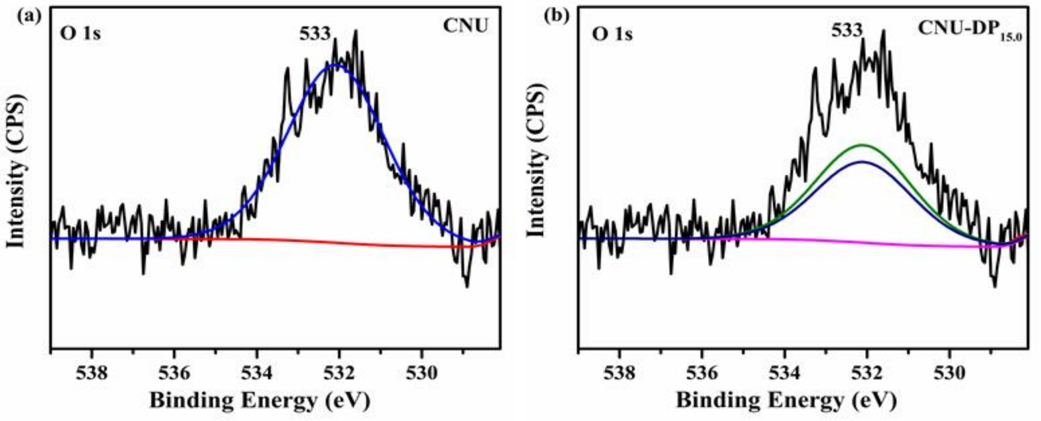

Figure S3. XPS results of high-resolution spectra of O 1s for CNU and CNU−$DP_{15.0}$.

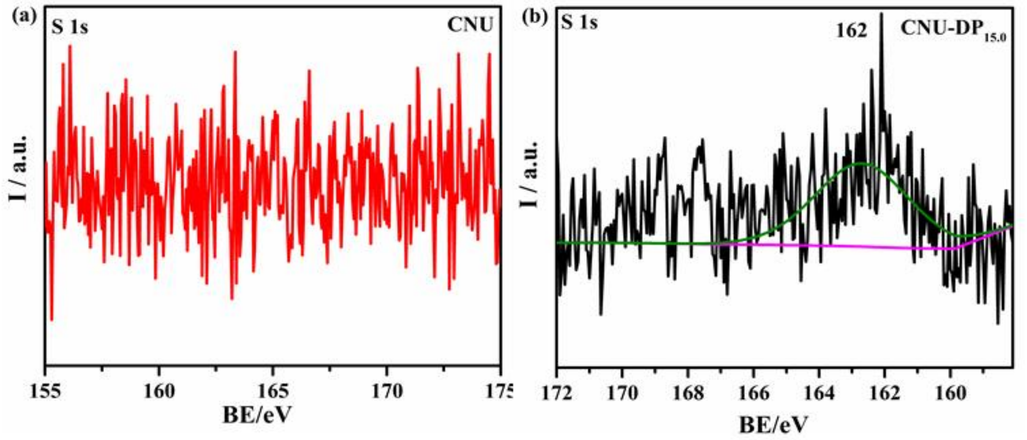

Figure S4.XPS results of high-resolution spectra of S 1s for CNU and CNU−$DP_{15.0}$.

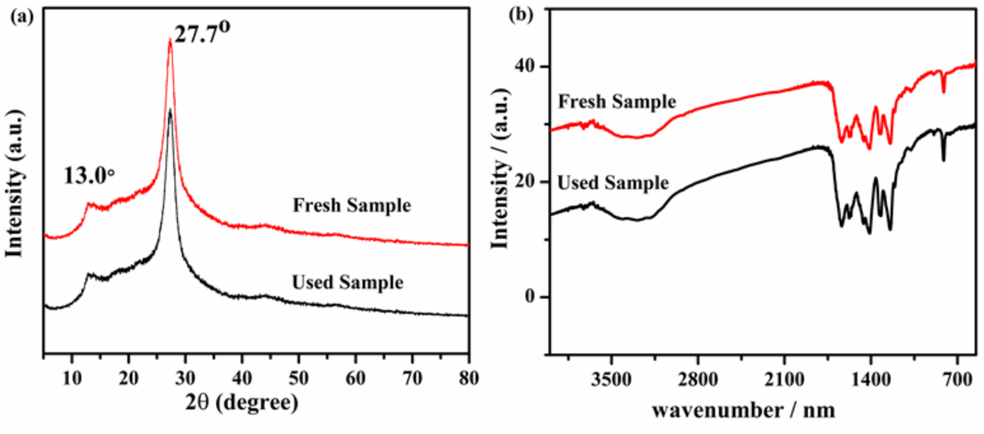

Figure S5. XRD **(a)** and FTIR **(b)** for fresh sample and used sample of CNU−$DP_{15.0}$.

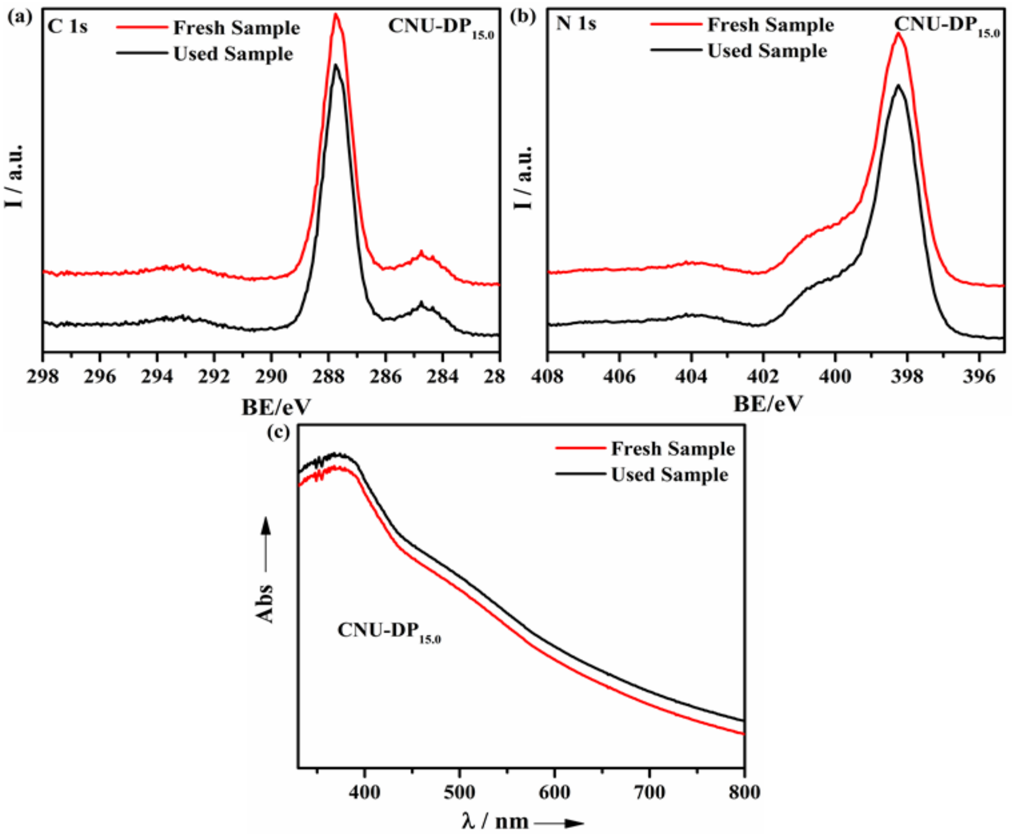

Figure S6.C 1s **(a)** and N 1s **(b)** UV-Vis DRS **(c)** for fresh sample and used sample of CNU−$DP_{15.0}$.

Table S5 The element derived by XPS results.

**Element**	**Atomic (W%)**
N	55.13
C	48.09
O	5.42
S	0.9

Table S6 Comparison of as-prepared samples with same class of co-monomers having different groups.

**Sample**	**Surface area[a] (m^2g^{-1})**	**Pore volume (cm^3 g^{-1})**	**Pore diameter (nm)**	**C/N molar ratio[b]**	**H_2[c] evolution (μmol h^{-1})**	**CO[c] evolution (μmol h^{-1})**
CNU	39.89	0.42	28.2	0.63	0.68	2.08
**CNU−$DP_{15.0}$**	**214.53**	**0.44**	**25.1**	**0.68**	**7.02**	**31.81**
CNU−$XT_{15.0}$	116.09	0.41	21.9	0.66	8.02	18.9
CNU−$CF_{15.0}$	112.55	0.45	23.6	0.66	5.48	15.2
CNU−$TB_{15.0}$	98.62	0.43	19.3	0.65	7.29	12.05

[a]Calculated from N_2 absorption–desorption isotherms.

[b]From elemental analysis spectra.

[c] From CO_2reduction controlled experiments.

Table S7. Study of various conditions on controlled experiments[a].

**Entry**	**CO(μmol)**	**H_2(μmol)**	**CO+H_2(μmol)**	**Sel.(%)[b]**
1	31.81	7.02	38.83	81.92
2[c]	n. d[d]	n. d	–	–
3[e]	n. d	n. d	–	–
4[f]	n. d	0.76	0.85	–
5[g]	n. d	0.59	–	–
6[h]	n. d	<0.1	<0.1	–
7[i]	n. d	<0.1	<0.1	–
8[j]	n. d	<0.1	<0.1	–
9[k]	n. d	0.30	0.25	–
10[l]	1.49	0.62	3.41	–
11[m]	3.01	1.57	3.50	–

[a] Reaction conditions: CNU−$DP_{15.0}$ (30mg), bpy (15mg), $COCl_2$ (1μmol), TEOA (1mL), solvent (MeCN 6mL), and λ>420nm, 30˚C,1 h.

[b] Selectivity=$n_{(CO)}/n_{(CO+H2)} \times 100$.

[c] Without CNU−$DP_{15.0}$

[d] Not determined.

[e] In dark.

[f] Using Ar, instead of CO_2.

[g] Without TEOA.

[h] Without bpy.

[i] Without $CoCl_2$.

[j] Without bpy and $CoCl_2$.

[k] Using N_2 instead of CO_2.

[l]Using of lactic acid as a sacrificial agent instead of TEOA.

[m] Using formic acid as a sacrificial agent instead of TEOA.

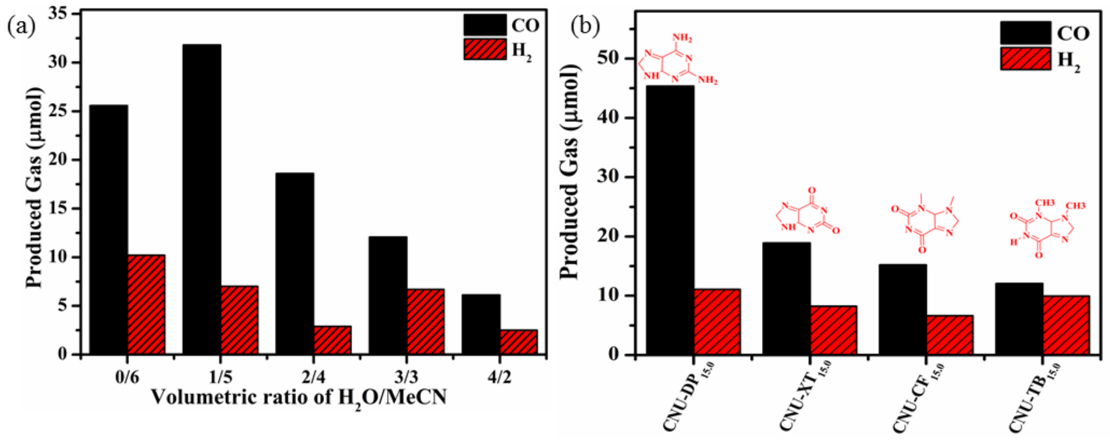

Figure S7. (a) Utiilizing CNU−DP$_{15.0}$ photocatalyst by using different volumetric ratio of water and acetonitrile **(b)** same class of monomer having different groups for the generation of CO and H_2 under visible light.

References

1. Yang, Z.; Mao, Z.; Zhang, X.; Ou, D.; Mu, Y.; Zhang, Y.; Zhao, C.; Liu, S.; Chi, Z.; Xu, J., Intermolecular Electronic Coupling of Organic Units for Efficient Persistent Room‐Temperature Phosphorescence. *Angewandte Chemie International Edition* **2016,** *55* (6), 2181-2185.
2. Hertwig, R. H.; Koch, W., On the parameterization of the local correlation functional. What is Becke-3-LYP? *Chemical Physics Letters* **1997,** *268* (5-6), 345-351.
3. Bauernschmitt, R.; Ahlrichs, R., Treatment of electronic excitations within the adiabatic approximation of time dependent density functional theory. *Chemical Physics Letters* **1996,** *256* (4-5), 454-464.
4. Rahman, M. U.; Wei, M.; Xie, F.; Khan, M., Efficient Dye-Sensitized Solar Cells Composed of Nanostructural ZnO Doped with Ti. *Catalysts* **2019,** *9* (3), 273.
